# Long-Term Outcomes of Intentional Head Trauma in Infants: A Comprehensive Follow-Up of Medical, Developmental, Psychological, and Legal Perspectives

**DOI:** 10.3390/medicina61020176

**Published:** 2025-01-21

**Authors:** Göksel Vatansever, Ezgi Özalp Akın, Pınar Bingöl Kızıltunç, Didem Behice Öztop, Kezban Karabağ, Seda Topçu, Betül Ulukol

**Affiliations:** 1 Department of Pediatrics, Ankara University School of Medicine, Dikimevi, 06620 Ankara, Türkiye; 2 Division of Developmental-Behavioral Pediatrics, Department of Pediatrics, Ankara University School of Medicine, 06620 Ankara, Türkiye; 3 Department of Ophthalmology, Ankara University School of Medicine, 06620 Ankara, Türkiye; 4 Department of Child and Adolescent Psychiatry, Ankara University School of Medicine, 06620 Ankara, Türkiye; 5 Division of Social Pediatrics, Department of Pediatrics, Ankara University School of Medicine, 06620 Ankara, Türkiye

**Keywords:** intentional head trauma 1, abuse 2, neglect 3

## Abstract

*Background and Objectives*: This study aimed to determine the initial clinical characteristics of children diagnosed with intentional head trauma (IHT) to obtain information about the long-term developmental, psychological, and psychosocial status of these children, to detect delayed sequelae, and to find out information about their judicial processes. *Materials and Methods*: Fourteen children who were followed up with the diagnosis of IHT in the Ankara Child Protection Unit between 2010 and 2021 were included in the study. These cases were evaluated in terms of physical, developmental, psychological, and visual findings. A complete physical examination was performed on the patients and their anthropometric measurements were taken. Anterior and posterior segment evaluations and visual field examinations were conducted in the visual assessment. The Expanded Guide for Monitoring Child Development and Vineland Adaptive Behavior Scale Third Edition was used in the developmental assessment. A psychiatric evaluation was performed using the Ankara Developmental Screening Inventory, Crowell observation, Affective Disorders and Schizophrenia Form, and Wechsler Intelligence Scale for Children. *Results*: Of the patients diagnosed with IHT, 71.4% were male and the mean age was 8.39 ± 5.86 (1.27–22.30; IQR: 3.55–11.96) months. In the long-term follow-up, cerebral palsy was detected in three of the children, epilepsy in one, optic atrophy and deviation due to this in one, and deviation due to brain trauma in one. Motor delay was detected in 50.0% of the patients, language delay in 37.5%, cognitive delay in 37.5%, and attention deficit and hyperactivity disorder in 25%. It was observed that the people who caused the injuries of two patients were punished. *Conclusions*: Children diagnosed with IHT should be monitored with transdisciplinary methods in terms of physical and mental health throughout childhood, starting from the first intervention. Awareness of IHT diagnosis should be increased with training in social service approaches and judicial authorities providing services for child neglect and abuse.

## 1. Introduction

Intentional head trauma (IHT) is a common form of child abuse that results in injury to intracranial structures or the skull due to violent shaking of the infant (acceleration and deceleration movements when the child is grabbed by the trunk or extremities and shaken forcefully), blunt impact, or a combination of both [1,2,3,4]. Studies have shown that the incidence varies between 14 and 40 per 100,000 [5,6,7]. If the child is not diagnosed when they first encounter it, there is a high probability that the child will be subjected to this abuse [8]. Most of the diagnosed babies may develop widespread axonal damage, subarachnoid hemorrhage, subdural hemorrhage, and retinal hemorrhage [9,10,11]. It is the most common cause of death due to child abuse [2]. Approximately 18–25% of the patients lose their lives and 75–80% experience long-term physical, neurological, and behavioral disorders due to neurological effects [1,8,12]. Therefore, due to brain damage and the effects of this damage on other areas, these children often show poor development and are at risk for poor school performance [13]. Long-term study data on the outcomes of these children are scarce due to sample sizes, variable follow-up periods, study designs, and the fact that most patients are lost to follow-up, and comprehensive studies are needed [13,14,15,16].

IHT is a type of maltreatment within the scope of child physical abuse. There are social, psychological, and legal implications for families [17]. As stated in Article 41 of the Constitution of the Republic of Türkiye, “The State shall take protective measures against all kinds of abuse and violence” and the protection of children from abuse and neglect is guaranteed by the state [18]. Therefore, when children with signs and symptoms suggestive of IHT apply to health service units for medical treatment, the person providing the health service must report the situation to the judicial authorities (Penal Code of Türkiye, Articles 280) [19]. In addition, the medical care needs of children monitored due to IHT are met in health institutions, and they are observed.

To be able to conduct studies on preventing abuse and healing children after diagnosis and to ensure that they continue their lives healthily, long-term outcomes need to be known. There is also a need to develop judicial and social service practices for the recovery of children who have been subjected to abuse and their participation in life appropriate to their age and culture. There are few studies in the literature investigating the long-term outcomes and developmental functioning of children. Some of these studies used administrative claims data from a comprehensive database and found developmental delays (motor deficits, communication disorders, and learning difficulties etc.), epilepsy, visual impairment, hearing loss, behavioral problems in long-term follow-up [15,16,20]. However, there is no research in the literature that examines the long-term physical and mental health, developmental, and legal outcomes of children with IHT with a holistic approach. This study aims to determine the initial clinical characteristics of children diagnosed with IHT, their long-term developmental, psychological, and psychosocial status, and outcomes, obtain information about their judicial processes, and evaluate the beneficial effects of legal monitoring for these children.

## 2. Materials and Methods

Children who were referred to the Ankara University Faculty of Medicine Child Protection Unit (ACPU) with a preliminary diagnosis of IHT from the outpatient clinics of the Department of Child Health and Diseases of Ankara University between January 2010 and January 2021 and who were evaluated by ACPU and legally notified with a diagnosis of IHT as a result of history, physical examination, and radiological [bone radiography, computed tomography (CT), and magnetic resonance imaging (MRI)] examinations were included in the study. The tools and examinations used in this study are summarized in Table 1.

The physical examination findings at the time of the IHT diagnosis and radiological examinations were evaluated by file review of the children. The families of these children were called and invited to have their children evaluated. The children of the families who accepted were evaluated. A comprehensive history of the children was taken and a physical examination (anthropometric measurements and complete systemic examination) was performed. Anthropometric measurements were assessed using growth reference curves for Turkish children [21].

The developmental assessments of the cases were conducted within the principles of the World Health Organization (WHO) International Classification of Functioning, Disability and Health (ICF), bioecological theory and the biopsychosocial model, using standard tools with a family-centered approach [33,34,35,36]. Additionally, a comprehensive medical and developmental history was obtained from the family and a thorough physical examination and observations of the child’s free play and child-family relationships were performed. The Expanded Guide for Monitoring Child Development (E-GMCD), Vineland Adaptive Behavior Scale Third Edition (Vineland-III), were utilized to assess development in all cases [22,23,37]. The open-ended questions of ‘‘Activities and participation” and ‘‘Environmental factors” sections of the Expanded Guide for Monitoring Child Development (Expanded GMCD), an ICF-based written questionnaire, were used to assess activities, participation, and environmental factors by the developmental pediatrician. For children aged 0 to 42 months, along with the Vineland-III, the GMCD and also Bayley Scales of Infant and Toddler Development, Third Edition (Bayley-III) were employed [22,23,25,37].

Vineland-III was used to assess adaptive development in four domains: communication (receptive, expressive, and written language subdomains), daily living skills (personal, community, and domestic daily living skills subdomains), socialization (interpersonal relationships, play and coping skills subdomains), and motor skills (fine and gross motor subdomains). To gather comprehensive information, interviews were conducted with families and, when necessary, teachers. The Adaptive Behavior Composite was obtained by summing the standard scores of the communication, daily living functions, and socialization domains. Subdomain scaled scores have a mean of 15 and a standard deviation of 3. The composite scores have a mean of 100 and a standard deviation of 15. When standard scores for core domains and adaptive functions were considered, <85 was considered mild and <70 was considered significant developmental delay; when scaled scores for subdomains (v-scale score) were considered, <12 was considered mild and <9 was considered significant developmental delay [23].

In the psychiatric evaluation, children between the ages of 0 and 3 were subjected to psychiatric interviews, developmental tests, Crowell observation, and clinical interviews based on DC: 0–5 [27]. Ankara Developmental Screening Inventory (AGTE) was applied to each child between the ages of 0 and 6 [26]. Affective Disorders and Schizophrenia Form for School-Age Children-Present and Lifetime Form DSM-5—Turkish Adaptation (ÇDŞG-ŞY-DSM-5-T) was applied to four patients between the ages of 6 and 12 [28,29,30]. In addition, the children’s intelligence levels were determined with the Wechsler Intelligence Scale for Children-Revised Form (WISC-R) [31]. The Specific Learning Disability Battery was applied to the patients who were considered to have a preliminary diagnosis of Specific Learning Disorder based on psychiatric and psychometric evaluation [32].

In the detailed ophthalmological examination of the patients, while visual acuity was evaluated as fixation and tracking pattern in the preverbal period, Snellen charts were used in the advanced age group. Eye movements and ocular alignment were evaluated as oculomotor functions. The degree of deviation was measured with the alternative prism coating test for distance and near. The Krimsky test was used in uncooperative patients. Color vision examination was performed with Ishihara charts and pupillary light reactions and afferent pupillary defects were evaluated. After examining the anterior segment, cycloplegic retinoscopy and fundus examination were performed.

Our study examined the legal reports of patients diagnosed with IHT. We requested information about the patients’ legal processes after diagnosis from local courts and evaluated the information received.

## 3. Results

**Figure 1:**
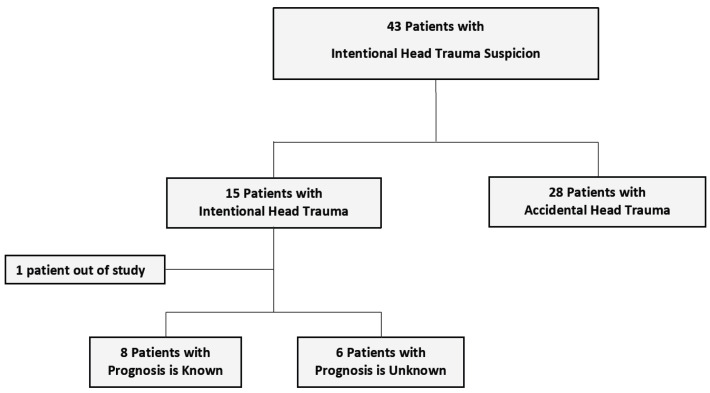
Distribution of patients evaluated with suspicion of intentional head trauma.

**Figure 2:**
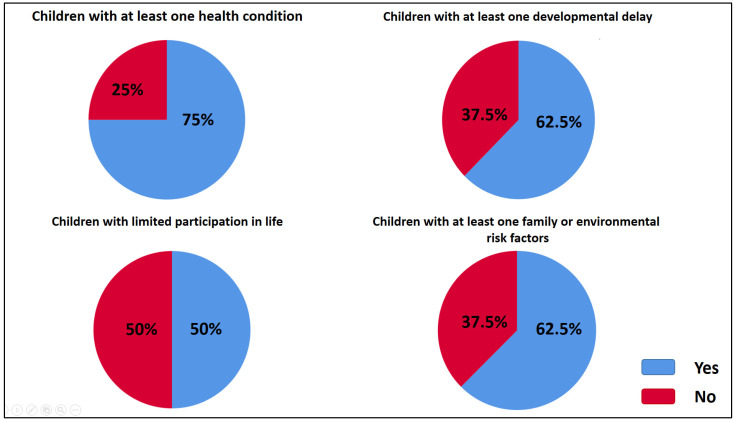
Frequency of children with intentional head trauma in the study in terms of health condition, developmental delay, limited participation in life, family/environmental risk factors (*n*:8).

Of the 43 patients evaluated by the ACPU with a preliminary diagnosis of intentional head trauma (IHT), 15 patients (32.5%) were confirmed to have IHT. One patient was excluded from the study due to inaccessible file data (see Figure 1). Characteristics and outcomes of the sample are summarized in Table 2 and Table 3 and Figure 2, while Table 4 and Table 5 present the median values of developmental assessment standard scores and composite scores, respectively.

### 3.1. Judicial Demographics

Judicial processes for the 14 patients diagnosed with IHT: information regarding the judicial processes of the 14 patients who were followed up with the diagnosis of IHT was requested from the relevant courts. While the local court ruled for non-prosecution in the files of nine (64.3%) patients, it was seen that indictments were prepared against the people who caused the incident in five (35.7%) patients, and two (14.3%) of them were sentenced. Health precautions were imposed on eight (57.1%) patients. One patient (7.1%) was given to a foster family and one patient (7.1%) was taken into institutional care. The other children (85.7%) were living with their families.

### 3.2. Patient Demographics

This article presents the data of 14 patients diagnosed with IHT whose file information was available. The mean age of children diagnosed with IHT was 8.39 ± 5.86 (1.27–22.30; IQR: 3.55–11.96) months. Of the patients, 10 (71.4%) were male and 4 (28.6%) were female.

Characteristics of children included in the study: The families of eight patients (57.1%) accepted check-ups; the remaining families refused participation. Six (75.0%) of the children were boys and two (25.0%) were girls. The mean age of the children was 65.66 ± 42.35 (14.90–123.30; IQR: 24.99–106.58) months. The median follow-up period, the time between diagnosis and evaluation, was 48.35 [IQR: 19.80–101.30] months.

### 3.3. Health Conditions

Four of the children (50%) had cerebral palsy, one child (12.5%) had epilepsy, and one child (12.5%) had optic atrophy in one eye. None of the children had malnutrition or stunting.

Visual Examination Results: In all but one of the eight patients who underwent eye examination, visual acuity was normal for their age group. The patient with low visual acuity had hand-movement level vision in one eye. Light reactions were normal in all patients and no afferent pupil defect was observed. In the evaluation of oculomotor function, all patients’ eye movements were free in all directions. Anterior segment structures were typical in all patients. In the retinal examination, atrophy in both optic discs, low vision, and misalignment secondary to optic atrophy were detected in one patient (12.5%). There was a misalignment that may be related to brain trauma in one patient.

### 3.4. Developmental and Mental Health Outcomes

Adaptive functioning based on Vineland-III assessment showed cognitive delay in three patients (37.5%) and language delay in three patients (37.5%). Cognitive development findings according to WISC-R showed that three patients (37.5%) were age-appropriate, three (37.5%) had borderline mental development, and two (25%) had moderate mental development. Attention deficit hyperactivity disorder was found in two patients (25%); a learning disability was found in one patient (12.5%). Psychiatric medication offered to ADHD-diagnosed patients; one patient refused medication. These findings indicate the need for individualized interventions and support to address the complex developmental and mental challenges faced by these children. In follow-up, an individualized intervention plan was created after determining the patients’ body structures, functions, personal and environmental factors, activity, and participation in life.

### 3.5. Family and Environmental Factors

It was found that all eight patients were under the supervision of the ACPU, with appropriate health measures in place. Regarding the educational background of the mothers and fathers, the majority completed high school education, while all were at least primary education graduates. Moreover, the monthly income of all families was above the minimum wage. Among the children, one (12.5%) was in institutional care, another in foster care, one (12.5%) lived with her mother, and the rest lived with their biological parents. Three children (37.5%) lacked sufficient nurturing care when evaluating the cases regarding responsive care and early learning opportunities. The mothers of two children (25%) reported depression in the Expanded GMCD.

### 3.6. Activities and Participation in Life

Most cases engaged in activities such as visiting relatives more than once a week, playing games with relatives’ children or siblings, spending time in parks, and being encouraged to participate in sports. Furthermore, a child in foster care played a musical instrument and participated in sports activities.

School-age distribution: Three children (37.5%) were not yet of school age. Three (37.5%) went to primary school and one (12.5%) went to kindergarten.

Special education services: One child (12.5%) was benefiting from individual special education, speech and language therapy, and rehabilitation services. In contrast, one child (12.5%) resided in institutional care and had not received individual special education, speech and language therapy, and rehabilitation services since 2019 due to the COVID-19 pandemic.

## 4. Discussion

Our study is the first one in Türkiye to reveal the long-term developmental outcomes of IHT patients based on the biopsychosocial model. Intentional head trauma is a condition that may cause serious health problems such as cranial and retinal hemorrhage and brain damage due to head trauma caused by physical abuse in early childhood and may even result in death. While it predominantly presents in children under the age of 2, its occurrence has been observed in children up to the age of 5, with the mean age of diagnosis being 6 months [38,39]. In our study, similar to the literature, the mean age at which children were diagnosed was 8.39 months, and 71.4% were male.

Few studies in the literature investigate the long-term outcomes and developmental functions of children diagnosed with IHT. The long-term consequences of children diagnosed with IHT have been reported mainly in high-income countries by scoring language and cognitive development and behavioral characteristics. Still, environmental factors, activities and participation in daily life, responsive care, and early learning opportunities of cases based on the biopsychosocial model have not been described [16,40,41]. Although the mortality and morbidity rates of IHT patients are not known exactly, the increase in awareness among professionals in recent years has increased the frequency of diagnosis [42]. Studies showing long-term outcomes in children report high morbidity rates (neurological sequelae, seizures, delay in language and cognitive development, behavioral problems, learning problems, delay in motor development, blindness, hearing impairment, nutritional deficiency) [16,20,40,41,43,44]. For instance, Barlow et al. prospectively evaluated the developmental and neurological outcomes in children with shaken baby syndrome with a mean age of 25.3 ± 9.1 months and cross-sectionally in another group with a mean age of 90 ± 50 months. The 5-year follow-up assessment revealed delays in motor development in 60% of the cases, vision problems in 48%, epilepsy in 20%, language difficulties in 64%, behavioral difficulties, and various difficulties in adaptive functions in 52% [40]. Similarly, Bonnier et al., in their longitudinal study of children with shaken baby syndrome, indicated that learning difficulties may not appear at an early age but may occur later in school years and suggested that these children be re-evaluated at this age [41]. The structures affected by IHT include ocular structures. Subconjunctival hemorrhage, periorbital edema/ecchymosis, corneal abrasion, hyphema, pupil anomalies, lens subluxation, cataracts, retinal findings, optic disc damage, globe rupture, and orbital fractures are the ocular effects that can be observed [45]. Retinal hemorrhage is the most common among retinal findings [46,47,48]. This study detected retinal hemorrhage, anisocoria, and periorbital ecchymosis as ocular findings at presentation. The most common ocular finding in patients was retinal hemorrhage in 46.7%. Ocular findings are observed bilaterally in 90% of patients with IHT, but involvement in both eyes may be asymmetrical [49]. Our study observed bilateral findings in all patients with ocular involvement. Ocular involvement in IHT may occur directly in the form of ocular structures being affected or developing indirectly due to intracranial structures being affected. As a result of the effects on ocular and intracranial structures, patients may have ophthalmic sequelae. In our study, some sequelae could be related to IHT in two patients, the first of which was due to optic nerve damage resulting from brain edema and axonal injury. The strabismus observed as a sequela in the second patient is also thought to be related to the subdural hematoma that occurred similarly. In our study, involving eight children diagnosed with IHT, long-term follow-up assessments were conducted using gold-standard methods within a family-centered, holistic, strength-based, transdisciplinary approach grounded in the International Classification of Functioning, Disability, and Health (ICF) and bioecological theory. The median age was 61.32 months (ranging from 14.90 to 123.30 months). Among these, three children had cerebral palsy; one had optic atrophy with associated deviation; another had deviation due to intracranial pathology; and three exhibited delayed adaptive functions.

Despite the small number of cases in the study, the presence of cognitive disability, learning difficulties, and attention problems in children are findings consistent with the literature. In contrast to the literature, there was a lower rate of language delay, cognitive delay, and neurological sequelae. It is not feasible to objectively assess the prevalence rate of the detected pathologies in the cases due to the families not accepting participation in the study. In addition, early mortality rates due to IHT may differ between studies. The fact that the cases with developmental difficulties and environmental and psychosocial risk factors in the sample showed improvement in their developmental functions in the follow-up after individualized intervention plans showed that these cases need long-term, comprehensive evaluations based on ICF, bioecological theory, and a biopsychosocial approach. Since IHT is a part of child neglect and abuse, early and long-term evaluation of these cases with the biopsychosocial model, determination of risk factors and protective factors, informing families, and their active participation in the intervention plan and follow-up with a family-centered holistic approach will prevent repeated abuse of the same child or abuse of other children in the family, and social costs will also decrease.

In line with our study, the most significant difficulties in studies on IHT have been reported to be the small number of patients, the high number of patients who dropped out of follow-up, and the lack of information on social outcomes. This may be related to families not wanting to continue their follow-up with the hospitals and doctors who diagnosed and reported IHT. A study reported positive development in only 8% to 32% of IHT cases that were followed up for more than 5 years [50]. The survey by Antoneitti et al. found that only 12.9% of patients underwent annual medical and neurodevelopmental evaluations and the follow-up period was approximately 2.5 years [51]. The absence of motor-neurological deficits and the acquisition of basic gross motor skills, such as sitting and walking, in the first few months following IHT may lead families to relax and abandon follow-up processes quickly. Indeed, in our study, the only child whose follow-up was continued in our clinic had severe motor-mental developmental deficits and was followed up with severe motor-cognitive insufficiency. In addition, the fact that only one of the eight patients in our study continued follow-up and that the families’ compliance with the recommended treatments was inadequate suggested that psychological support was not received and that they were medically neglected as a result.

To monitor IHT and prevent recurrence in the patient or siblings, it is necessary to determine the risk and protective factors related to the child, family, and environment that play a role in child neglect and abuse and to plan the intervention plan within this framework [52]. A study examining the long-term security and social status of IHT patients was encountered, in which only the medical and social results of 80 cases diagnosed with IHT were retrospectively reviewed, 50 (64.9%) returned home, and 19 (24.7%) were given to foster care [51]. All of our patients participating in the study were under monitoring by the ACPU; all patients were given health precautions, one patient (12.5%) was given to a foster family, one patient (12.5%) was taken to institutional care, and the other children were living with their families. Families of children not placed on health measures by the court did not agree to participate in the study.

Child abuse is a crime and has several social and legal consequences. However, for years, the diagnosis of IHT has been approached with skepticism among the medical and legal communities. This has led to different opinions among physicians, different evaluations among judges, and, in some cases, cases that cannot be proven beyond doubt, resulting in differences in legal decisions [53]. In our study, it was seen that the local court indicted 35.7% of the people who caused the incident and 14.3% were sentenced. Health precautions were placed on 57.1% of the patients. This situation may be because judges may decide in favor of accidental injury when evaluating the incident if the child is not seriously injured and they do not think that follow-up is necessary, or it may result from the difficulty of proving beyond doubt in many cases. Differences in evaluation can be reduced by increasing the number of professionals who have received special training in intervention methods related to social service approaches by establishing the necessary legal regulations and local courts specialized in IHT and other child neglect and abuse.

Non-compliance with follow-up recommendations poses a significant barrier to the effective management and rehabilitation of IHT patients. Factors contributing to non-compliance may include psychological distress, lack of awareness about the importance of follow-up, socioeconomic challenges, and fear of legal repercussions. Our findings imply that there is a need for action by clinicians, researchers, and policymakers to provide long-term follow-up based on bioecological theory on a child’s health, development, and participation in life as well as the child’s living conditions, family, and environment for children with IHT. Governments should implement family-centered, transdisciplinary, long-term follow-up models.

The fact that some of the families of the patients invited to the study did not accept to participate in the study and that the follow-up results of all patients are not fully known constitutes a limitation of the study. The small number of patients participating in the study may have created bias in the evaluation of the frequency of the findings. Therefore, future studies should be conducted with larger samples to ensure the generalizability of the findings. Multicenter studies can increase the number of participants and representativeness, especially in countries with scarce data. Furthermore, new research examining the role of environmental (familial, social, etc.) factors and different intervention models on children with IHT will contribute to comprehensive interventions and improve the quality of life of affected children.

## 5. Conclusions

Intentional head trauma presents a multitude of risks and potential scenarios. Our investigation reveals that non-adherence to recommended follow-up and treatments by families indicates a necessity for implementing comprehensive health precautions throughout childhood, as there is a risk of medical neglect associated with this condition. These children’s physical and mental health, development, and well-being should be monitored by different disciplines with transdisciplinary methods holistically throughout childhood, starting from the first intervention. During this period, it is essential to determine risk factors with family-centered and individualized assessments based on the biopsychosocial model, implement timely protective interventions, and provide social support for the child and family. The successful implementation of this initiative with a family-centered holistic approach, involving the active participation of the family throughout this process, will contribute to reducing societal costs by preventing the recurrence of the child’s abuse or the abuse of other children within the family.

## Figures and Tables

**Table 1:** The tools and examinations used in this study.

Tool/Examination Method	Description	Rationale for Selection
Antropometric charts [21]	Growth reference curves that assess the physical growth of children based on age and sex, specifically for Turkish children.	These charts are crucial for assessing physical growth patterns in infants. In cases of IHT, abnormal growth may indicate neglect or abuse. Monitoring growth trajectories helps identify potential health issues related to trauma or malnutrition, which are often associated with abusive environments.
Visual assessment	An evaluation of visual acuity and eye function using various methods, including Snellen charts and fixation patterns.	Visual assessments are essential for detecting potential ocular injuries resulting from trauma. Research indicates that visual impairments can be a consequence of head trauma, particularly in cases of abuse, where injuries may not be immediately apparent. Early identification allows for timely intervention to prevent long-term visual deficits.
The Expanded Guide for Monitoring Child Development [22]	A comprehensive WHO-ICF-based tool that assesses a child’s health condition, developmental condition, activities and participation in life, and environmental context through caregiver knowledge.	Holistic assessment of each child and family.
Vineland Adaptive Behavior Scale Third Edition (Vineland- III) [23,24]	A standardized widely used assessment measuring adaptive behavior across communication, daily living skills, socialization, and motor skills.	Evaluation of adaptive functioning, guidance for targeted interventions.
Bayley Scales of Infant and Toddler Development, Third Edition (Bayley-III) [25]	The Bayley-III is specifically designed to assess cognitive, language, and motor development in children aged 1 to 42 months.	By providing a structured framework for assessing developmental functioning, the Bayley-III helps healthcare providers formulate individualized intervention plans tailored to each child’s specific needs.
Ankara Developmental Screening Inventory [26]	A screening tool designed to assess developmental milestones in children aged 0–6 years.	This screening tool is specifically designed for young children and is vital for detecting developmental milestones that may be affected by trauma. It ensures that any delays are recognized early, allowing for timely support and intervention.
Crowell observation [27]	A method involving structured observation of child behavior during play to assess emotional and social development.	Structured observations during play provide insights into the child’s emotional and social development. Given that IHT can significantly impact these areas, such observations help clinicians understand the child’s behavior in a natural context, revealing potential emotional disturbances linked to trauma.
Affective Disorders and Schizophrenia Form [28,29,30]	A standardized form for assessing affective disorders in school-age children based on DSM-5 criteria.	This form aids in assessing psychological conditions in children aged 6–12 years who may have experienced trauma. Identifying affective disorders is crucial as they can complicate recovery and development following IHT.
Wechsler Intelligence Scale for Children [31]	A widely used intelligence test designed to measure cognitive ability in children.	The WISC-R provides a measure of cognitive ability, which is essential for understanding how IHT may affect learning and intellectual development. Cognitive assessments can reveal deficits that need to be addressed through educational interventions.
The Specific Learning Disability Battery [32]	A set of assessments designed to evaluate specific learning disabilities in children.	This battery identifies specific learning disabilities that may arise in children who have experienced IHT. Early detection allows for tailored educational strategies to support learning and development in affected children.

**Table 2:** Demographic characteristics, legal outcomes, and prognoses of patients diagnosed with intentional head trauma.

Case	Age (Mo)	Gender	Symptoms	Clinical Findings	Radiological Findings			Ocular Findings	Risk Factors	Juridical Process	Prognosis
					Bone X-ray	Cranial CT	Cranial MR				
1	9.5	F	Swelling on the head	N	Parietal bone fracture	EDH SDH	-	N	Delayed hospital application Low socioeconomic status	Non-prosecution	Specific learning disability
2	6.2	M	Seizure	N	N	SDH	-	N	Mother and father not officially married Father in prison	Non-prosecution	No psychopathology detected
3	11.8	F	Projectile vomiting and weakness	Ecchymosis on face Ecchymosis on chest Swelling in extremities	Occipital bone, clavicle, radius fracture	CE	DAI	BRH	Low socioeconomic status Lives with mother and stepfather; mother and stepfather are not officially married.	Indictment (the father punished)	Spasticity Vision loss
4	3.9	M	Weakness	Ecchymosis on face Ecchymosis on extremities	N	SDH	DAI	BRH	Twin pairs Mother is the sole caregiver Lack of social support The twin pair has a congenital anomaly	Indictment	ADHD Borderline intellectual functioning
5	2.6	M	Projectile vomiting and weakness	Ecchymosis on face	N	SDH	DAI	BRH	Infantile colic Mother works during the day; father works at night Father looks after child sleeplessly during the day	Indictment	ADHD Borderline intellectual functioning Spasticity
6	23.3	M	Seizure and loss of consciousness	Unconsciousness	N	SAH	DAI SDH	BRH	Delayed hospital apply Father in prison Mother was raised in an institution Surgery due to aortic coarctation	Indictment	Moderate cognitive disabilities Moderate mental development Spasticity
7	2.1	M	Cardiac arrest	Unconsciousness Anisocoria Respiratory arrest	N	SDH	SDH	BRH	Infantile colic	Non-prosecution	Moderate cognitive disabilities Moderate mental development Spasticity
8	7.0	M	Seizure	N	N	SDH	SDH	BRH	Prematurity	Non-prosecution	No psychopathology detected
9	13.7	M	Seizure	N	N	-	EDH	BRH	Low socioeconomic status	Non-prosecution	Unknown
10	0.8	F	Swelling on the head	N	-	-	-	N	The presence of a special needs individual in the family	Non-prosecution	Unknown
11	8.6	M	Swelling on the head	Hematoma on head	Parietal bone fracture	EDH	-	-	Delayed hospital application	Non-prosecution	Unknown
12	10.5	F	Seizure	Ecchymosis on abdomen	N	IVH	-	-	Young mother Psychiatric illness in mother	Indictment (the mother punished)	Unknown
13	4.6	M	Seizure	N	N	-	DAI SDH	N	-	Non-prosecution	Unknown
14	12.4	F	Fallen on	Sunset eyes	N	SDH	DAI SDH	N	Low socioeconomic status The presence of a special needs individual in the family	Non-prosecution	Unknown

Attention-deficit/hyperactivity disorder: ADHD; bilateral retinal hemorrhages: BRH; cerebral edema: CE; diffuse axonal injury: DAI; epidural hematoma: EDH; female: F; intraventricular hemorrhage: IVH; male: M; normal: N; subarachnoid hemorrhage: SAH; subdural hematoma: SDH.

**Table 3:** Characteristics of the sample within the scope of bioecological theory.

Characteristics of the Children (Health, Developmental Status, Participation in Life)	*n*:8	%
**Children’s health status**		
Prematurity	1	12.5
Cerebral palsy	4	50.0
Epilepsy	1	12.5
Optic atrophy	1	12.5
Malnutrition/stunting	0	0
B12 deficiency	1	12.5
**Children’s developmental status**		
Delay in adaptive function	2	25.0
Significant delay in adaptive function	1	12.5
Visual impairment	1	12.5
Attention-deficit/hyperactivity disorder	2	25.0
Eating problem	0	0
**Children’s participation in life**		
Less than once a week;		
Visiting relatives	3	37.5
Playing with other children	2	25.0
Playing with animals or in nature	2	25.0
Sports and hobbies	2	25.0
Going to kindergarten (*n* = 5, >3 years)	1	20.0
Receiving special education and rehabilitation services	1	12.5
**Family and environmental factors**		
Mother’s education ≤ high school	6	75.0
Father’s education ≤ high school	5	62.5
Monthly income < minimum wage	0	0
Reported depression in the mother	2	25.0
Divorce or family conflict	2	25.0
Institutional care/living in a foster family	2	25.0
Single parent	1	12.5
Inadequacy of nurturing care	3	37.5

**Table 4:** Assessment results of adaptive functions.

Vineland-III Developmental Domains and Sub-Domains	Median	Minimum	Maximum
**Communication domain standard score**	93	30	109
v-Scaled score of receptive language subdomain	12.5	6	18
v-Scaled score of expressive language subdomain	14	1	18
v-Scaled score for written language subdomain	11	1	16
**Daily living skills domain standard score**	106	41	134
v-Scaled score of personal subdomain	18	1	23
v-Scaled score of domestic subdomain	15	5	20
v-Scaled score of community subdomain	14	3	19
**Socialization domain standard score**	100	61	123
v-Scaled score of interpersonal relationships subdomain	16	5	20
v-Scaled score of play and leisure subdomain	14.5	10	19
v-Scaled score of coping skills subdomain	14	8	18
**Motor domain standard score**	103	59	124
v-Scaled score of gross motor subdomain	17	5	22
v-Scaled score of fine motor subdomain	16	3	19
**Adaptive behavior composite**	96.5	44	127

**Table 5:** Evaluation of the results of the developmental functions in children younger than 42 months (*n*:3) in the sample.

Bayley-III Developmental Domains	Median	Minimum	Maximum
Cognitive composite score	85	85	105
Language composite score	86	71	91
Motor composite score	94	88	100

## Data Availability

Due to legal restrictions, the data presented in this study cannot be shared directly. Data sharing will be based on the specific request.
